# Supplementary material for: Tuning Electromagnetic Parameters Induced by Synergistic Dual-Polarization Enhancement Mechanisms with Amorphous Cobalt Phosphide with Phosphorus Vacancies for Excellent Electromagnetic Wave Dissipation Performance
Source: Nanomaterials (Basel). 2023 Nov 27;13(23):3025. doi: 10.3390/nano13233025 (PMC10708327; doi:10.3390/nano13233025)
Supplement: Supplementary file 1 [file nanomaterials-13-03025-s001.zip › nanomaterials-2703542-supplementary.pdf]

nanomaterials

Supporting Information

Tuning Electromagnetic Parameters Induced by Synergistic Dual-Polarization Enhancement Mechanisms with Amorphous Cobalt Phosphide with Phosphorus Vacancies for Excellent Electromagnetic Wave Dissipation Performance

Bo Wen [1,2,3], Yunzi Miao [1,2,3], Zhijie Zhang [4], Na Li [1,2,3], Jiyuan Xiao [1,2,3], Yushuo Li [1,2,3], Jiangtao Feng [4], Shujiang Ding [1] and Guorui Yang [1,2,3,*]

1. School of Chemistry," Xi'an Jiaotong University, Xi'an 710049, China; wenbom@stu.xjtu.edu.cn (B.W.); myznofwplz@163.com (Y.M.); lina0831@xjtu.edu.cn (N.L.); xjy@nwafu.edu.cn (J.X.); lys11544@163.com (Y.L.); dingsj@mail.xjtu.edu.cn (S.D.)
2. Engineering Research Center of Energy Storage Materials and Devices, Ministry of Education, "Four Joint Subjects One Union，Xi'an Jiaotong University, Xi'an 710049, China
3. School-Enterprise Joint Research Center for Power Battery Recycling & Circulation Utilization Technology, Xi'an Jiaotong University, Xi'an 710049, China
4. Department of Environmental Science and Engineering, Xi'an Jiaotong University, Xi'an 710049, China; zzhijie@stu.xjtu.edu.cn (Z.Z.); fjtes@xjtu.edu.cn (J.F.)

* Correspondence: yangguorui@xjtu.edu.cn.

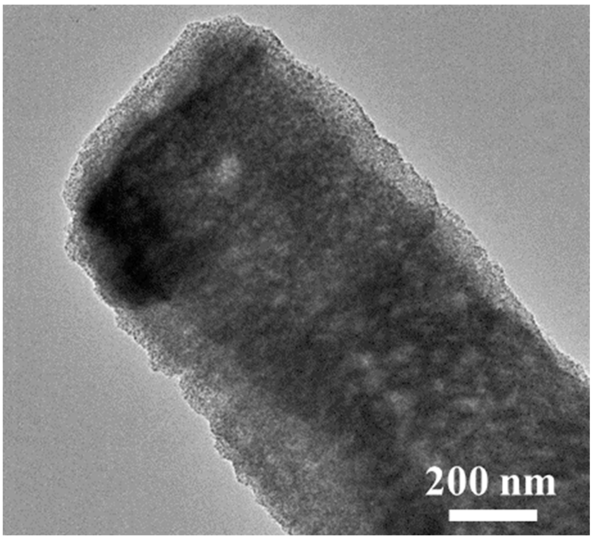

Figure S1. TEM images of the Co@C composites.

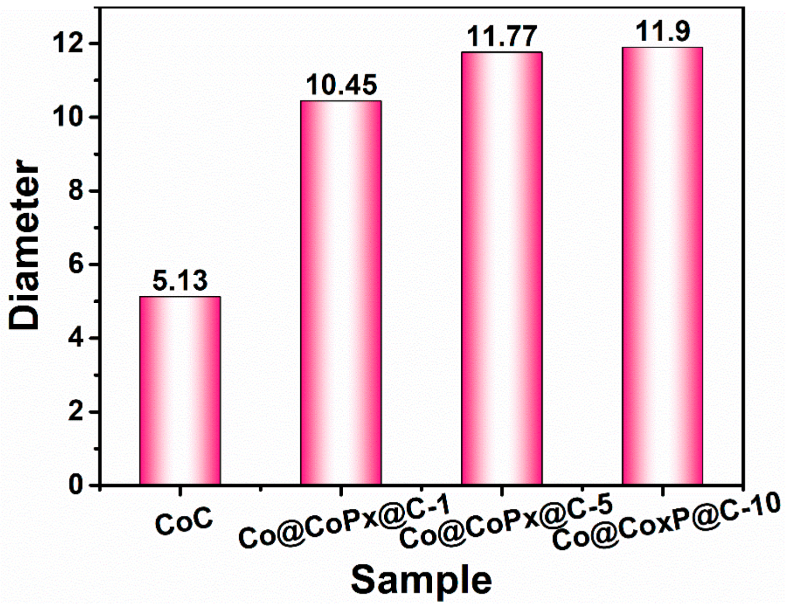

Figure S2. The average particle sizes of Co@C, Co@CoPx@C-1, Co@CoPx@C-5 and Co@CoPx@C-10 composites.

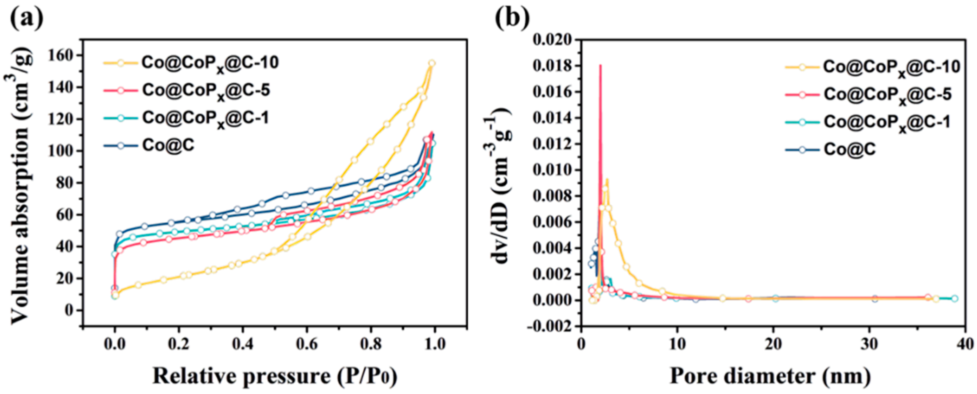

Figure S3. (a) Nitrogen adsorption/desorption isotherms and **(b)** the corresponding pore size distribution of Co@C, Co@CoPx@C-1, Co@CoPx@C-5 and Co@CoPx@C-10 composites.

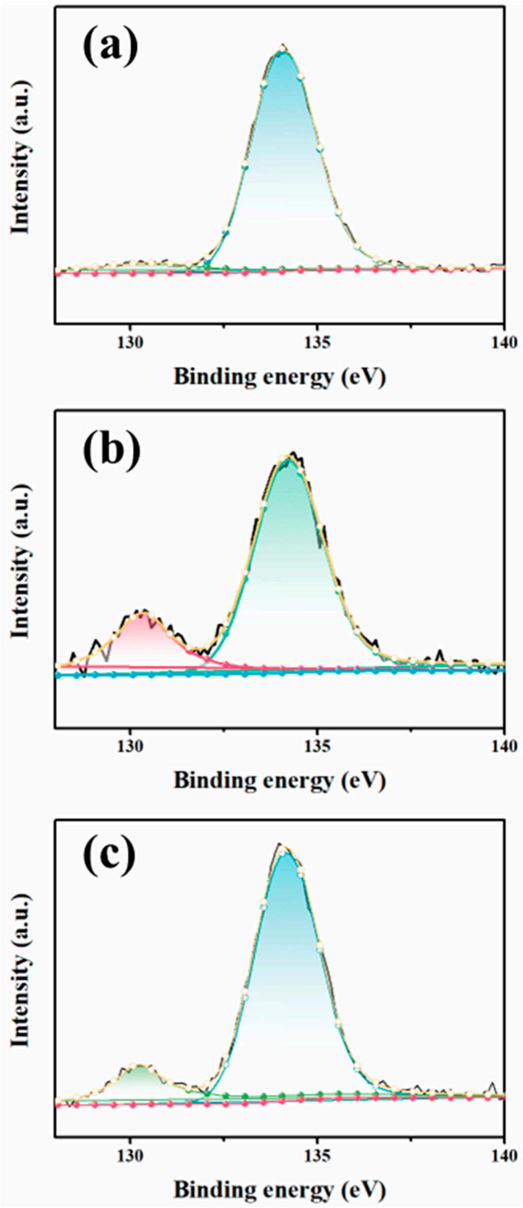

Figure S4. high-resolution P 2p of **(a)** Co@CoP$_x$@C-1, **(b)** Co@CoP$_x$@C-5 and (c) Co@CoP$_x$@C-10 composites.

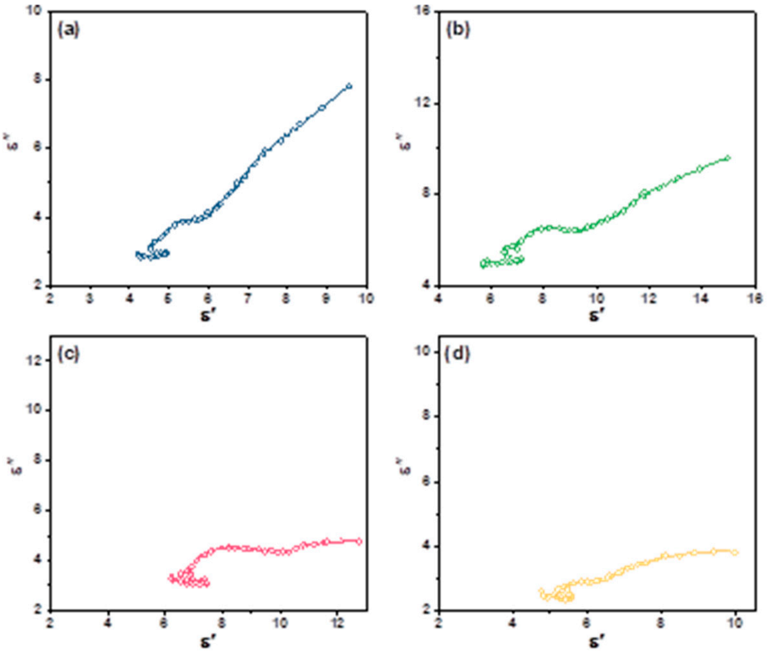

Figure S5. Cole-Cole curves for the **(a)** Co@ Co@C, **(b)** Co@CoPx@C-1, **(c)** Co@CoPx@C-5 and **(d)** Co@CoPx@C-5 composites.

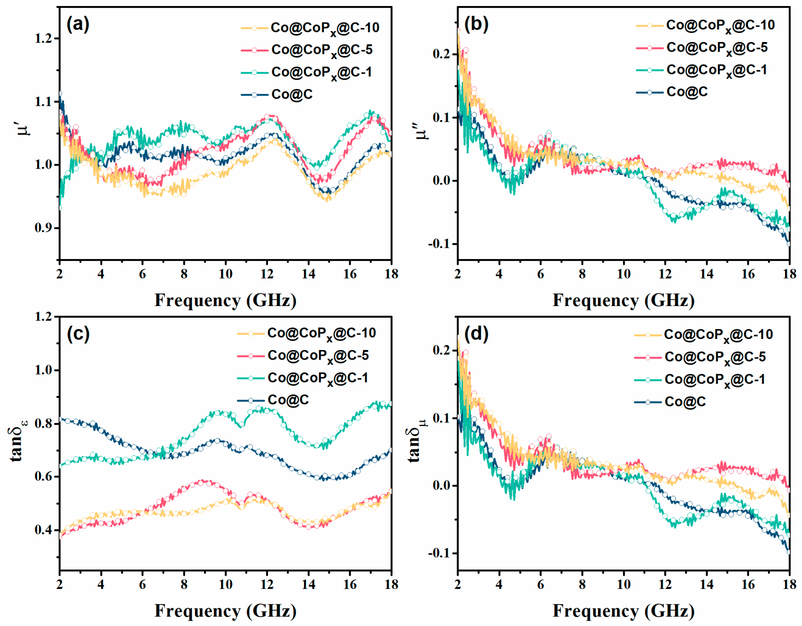

Figure S6. **(a)** real parts μ′, **(b)** imaginary parts of complex permeability, **(c)** dielectric loss tangent ($tan\delta_\varepsilon$) and **(d)** magnetic loss tangent ($tan\,\delta_\mu$) of Co@C, Co@CoPx@C-1, Co@CoPx@C-5 and Co@CoPx@C-10 composites.

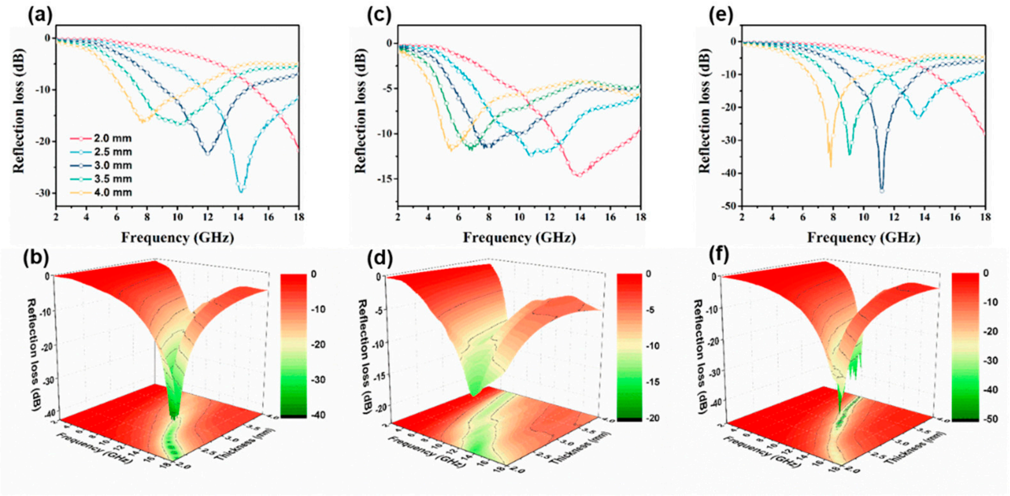

Figure S7. 2D RL curves and 3D plots of **(a,b)** Co@C, **(c,d)** Co@CoP$_x$@C-1 and **(e,f)** Co@CoP$_x$@C-10 composites.

Table S1. Relevant electromagnetic fitting parameters of the samples.

Samples	ε_s	ε_∞	σ (S/M)	τ
Co@C	80.66	4.021	0.59721	0.000132
Co@CoP@C-1	102.865	1.335	0.52602	0.000244
Co@CoP@C-5	85.408	1.659	0.50293	0.000266
Co@CoP@C-10	58.009	2.587	0.42595	0.000146
